# Characterization of blood dendritic and regulatory T cells in asymptomatic adults with sub-microscopic *Plasmodium falciparum* or *Plasmodium vivax* infection

**DOI:** 10.1186/s12936-016-1382-7

**Published:** 2016-06-21

**Authors:** Steven Kho, Jutta Marfurt, Irene Handayuni, Zuleima Pava, Rintis Noviyanti, Andreas Kusuma, Kim A. Piera, Faustina H. Burdam, Enny Kenangalem, Daniel A. Lampah, Christian R. Engwerda, Jeanne R. Poespoprodjo, Ric N. Price, Nicholas M. Anstey, Gabriela Minigo, Tonia Woodberry

**Affiliations:** Global and Tropical Health Division, Menzies School of Health Research and Charles Darwin University, Darwin, Australia; Eijkman Institute for Molecular Biology, Jakarta, Indonesia; Timika Malaria Research Programme, Papuan Health and Community Development Foundation, Timika, Papua Indonesia; Rumah Sakit Umum Daerah Kabupaten Mimika, Timika, Papua Indonesia; QIMR Berghofer Medical Research Institute, Brisbane, Australia; Department of Paediatrics, University of Gadjah Mada, Yogyakarta, Indonesia; Nuffield Department of Medicine, Centre for Tropical Medicine and Global Health, University of Oxford, Oxford, UK

**Keywords:** Malaria, Clinical immunity, Dendritic cell, Regulatory T cell, Asymptomatic, Sub-microscopic, Adults, Vivax, Falciparum, Human

## Abstract

**Background:**

*Plasmodium falciparum* and *Plasmodium vivax* infections compromise dendritic cell (DC) function and expand regulatory T (Treg) cells in both clinical disease (malaria) and experimental human sub-microscopic infection. Conversely, in asymptomatic microscopy-positive (patent) *P. falciparum* or *P. vivax* infection in endemic areas, blood DC increase or retain HLA-DR expression and Treg cells exhibit reduced activation, suggesting that DC and Treg cells contribute to the control of patent asymptomatic infection. The effect of sub-microscopic (sub-patent) asymptomatic *Plasmodium* infection on DC and Treg cells in malaria-endemic area residents remains unclear.

**Methods:**

In a cross-sectional household survey conducted in Papua, Indonesia, 162 asymptomatic adults were prospectively evaluated for DC and Treg cells using field-based flow cytometry. Of these, 161 individuals (99 %) were assessed retrospectively by polymerase chain reaction (PCR), 19 of whom had sub-microscopic infection with *P. falciparum* and 15 with sub-microscopic *P. vivax* infection. Flow cytometric data were re-analysed after re-grouping asymptomatic individuals according to PCR results into negative controls, sub-microscopic and microscopic parasitaemia to examine DC and Treg cell phenotype in sub-microscopic infection.

**Results:**

Asymptomatic adults with sub-microscopic *P. falciparum* or *P. vivax* infection had DC HLA-DR expression and Treg cell activation comparable to PCR-negative controls. Sub-microscopic *P. falciparum* infection was associated with lower peripheral CD4^+^ T cells and lymphocytes, however sub-microscopic *Plasmodium* infection had no apparent effect on DC sub-set number or Treg cell frequency.

**Conclusions:**

In contrast to the impairment of DC maturation/function and the activation of Treg cells seen with sub-microscopic parasitaemia in primary experimental human *Plasmodium* infection, no phenotypic evidence of dysregulation of DC and Treg cells was observed in asymptomatic sub-microscopic *Plasmodium* infection in Indonesian adults. This is consistent with DC and Treg cells retaining their functional capacity in sub-microscopic asymptomatic infection with *P. falciparum* or *P. vivax* in malaria-endemic areas.

**Electronic supplementary material:**

The online version of this article (doi:10.1186/s12936-016-1382-7) contains supplementary material, which is available to authorized users.

## Background

A significant proportion of individuals living in areas where malaria is endemic have *Plasmodium* parasitaemia without the presence of symptoms. This is thought to reflect either the early stages of infection or the acquisition of clinical immunity that restricts parasite expansion and prevents clinical disease [1, 2]. Microscopy or rapid diagnostic tests remain the most common method for the diagnosis of *Plasmodium* infection. However, it is now recognized that even in low-transmission settings a large proportion of microscopy-negative individuals have sub-microscopic parasitaemia identified by polymerase chain reaction (PCR) [3–7]. These sub-microscopic infections frequently remain undetected and untreated and hence, contribute significantly to the transmittable reservoir and potentially to morbidity [8]. Sensitive molecular techniques are now used increasingly in epidemiological studies to detect sub-microscopic infection with limits of detection below 0.1 parasites per μl [9–12]. Sub-microscopic *Plasmodium* infections are important and warrant consideration in malaria intervention and elimination programmes [9, 13].

While immune correlates in microscopy-positive asymptomatic parasitaemia have been reported [14–17], few data exist on immune regulation at very low parasitaemia, with no data available on the dendritic cell (DC) and regulatory T (Treg) cell response at sub-microscopic levels of infection in malaria-endemic areas. In individuals hospitalized with clinical disease from *Plasmodium falciparum* and *Plasmodium vivax*, blood DC are functionally and numerically compromised [18, 19] while Treg cells expand [20, 21], promoting immune suppression. Similarly, in low-dose experimental *P. falciparum* blood-stage infection of naïve volunteers, blood DC become non-functional and apoptose at PCR-level parasitaemia below the detection limit of microscopy [22, 23]. In contrast, the recent demonstration that adults and children with asymptomatic microscopy-positive *P. falciparum* or *P. vivax* infection have increased or retained activation of blood DC and reduced activation of Treg cells [14], suggests that appropriate DC maturation and less Treg suppression may contribute to the active control of asymptomatic infection. Therefore, in contrast to naïve individuals with sub-microscopic infection, it is hypothesized that asymptomatic-exposed individuals with sub-microscopic infection respond similarly to patent asymptomatic-exposed individuals, such that DC remain functional while Treg cells display reduced suppressive function.

In a previous publication [14], parasitaemia in asymptomatic adults was evaluated by microscopy only. Using PCR, a large proportion of microscopy-negative controls were subsequently identified as having sub-microscopic *Plasmodium* infection. In an extension of the original study [14], flow cytometry data were re-categorized in adults using the additional PCR results. Sub-microscopic infection with *P. falciparum* or *P. vivax* was identified from microscopy-negative controls and then compared to patent infection and acute malaria. The results indicate that while peripheral CD4^+^ T cells decline, blood DC and Treg cells remain numerically and phenotypically, functionally preserved during asymptomatic sub-microscopic *P. falciparum* or *P. vivax* infection.

## Methods

### Study participants and sample collection

The field study was conducted in Timika, in Papua, Indonesia, a region with high but unstable malaria transmission [24]. Participants were enrolled and sampled as part of a household survey conducted in 2013, as previously reported [14]. Briefly, 162 Papuan and non-Papuan adults had venous blood taken, were diagnosed for *Plasmodium* infection by microscopy, and were evaluated by field-based flow cytometry of peripheral blood for DC and Treg cells [14]. All participants were asymptomatic with no documented fever (<37 °C), or other symptoms of malaria at recruitment or within the preceding 24 h. Participants with detectable parasitaemia by microscopy were treated with dihydroartemisinin–piperaquine according to local protocols. Approximately 200 µl of packed red blood cells (RBCs) were frozen per adult to verify patent infections and identify sub-microscopic *Plasmodium* infections by PCR. As a comparator group, 14 adults being treated for acute uncomplicated malaria at Rumah Sakit Mitra Masyarakat (RSMM) hospital in Timika were enrolled as described previously [14].

### Identification of sub-microscopic infection

Frozen RBCs were transferred to the Eijkman Institute for Molecular Biology in Jakarta for molecular analysis. The QIAamp 96 DNA Blood Kit (Qiagen) was used according to manufacturer’s instructions to extract genomic DNA from >50 µl of RBCs. Gene target amplification for *P. falciparum*, *P. vivax*, *Plasmodium malariae*, and *Plasmodium ovale* speciation was performed on a Veriti 96-W thermal cycler (Thermo Fisher Scientific) using a nested PCR approach as described elsewhere [25]. The limit of detection of the assay was 0.2 parasites per µl as evaluated using well-characterized samples of *P. falciparum* and *P. vivax* (unpublished observations: Piera et al.). Positive results reflected live parasites, with previous clinical and human challenge studies showing that dead or dying parasites are cleared within one to 3 days of anti-malarial treatment [26, 27].

### Whole blood flow cytometry

DC and Treg cells were identified by fresh whole blood flow cytometry using protocols detailed previously [14]. DC and Treg antibody panels are listed in Additional file 1. All samples were acquired on a portable BD Accuri C6 flow cytometer using CFlow Sampler Software (BD Biosciences). All antibodies were purchased from BioLegend (San Diego, CA, USA).

### Data analysis

Asymptomatic individuals were categorized into sub-microscopic or patent infection according to microscopy and PCR results. Individuals that were both microscopy- and PCR-negative were considered uninfected controls. Those that were microscopy-negative and PCR-positive were categorized as sub-microscopic infections. Microscopy-positive individuals were considered as patent infections regardless of their PCR results. Flow cytometric data were analysed on FlowJo (TreeStar, Ashland, OR, USA) and absolute calculations performed as described elsewhere [14]. DC and Treg cell flow cytometry data from the same sample were only available in 41 % of participants due to either constraints in the amount of blood available for testing, or loss of sample quality due to insufficient lysis of RBCs during processing. Samples with fewer than 20 events in the DC sub-set gates were excluded. All statistical analyses were conducted using GraphPad Prism 6 (GraphPad Software, La Jolla, CA, USA). The Kruskal–Wallis test with Dunn’s multiple comparisons test, or the Mann–Whitney U test was used for statistical comparison as indicated.

### Ethics

The study was approved by the Human Research Ethics Committees of Gadjah Mada University, Yogyakarta, Indonesia, the Eijkman Institute Research Ethics Commission, Jakarta, Indonesia, and the NT Department of Health and Families and Menzies School of Health Research, Darwin, Australia. Written informed consent was obtained from all participants (or the primary caregiver or relative) prior to blood sampling.

## Results

### Study population

Of the 162 asymptomatic adults that were originally sampled, 122 were microscopy-negative and 40 had patent infections. One microscopy-negative individual had no PCR data and was excluded. Thirty-one per cent (38/121) of the microscopy-negative adults had sub-microscopic infection with *Plasmodium*. Four sub-microscopic infections were excluded from analysis due to either mixed, or *P. malariae* sub-microscopic infection. A further four adults with mono-species infection by microscopy were excluded due to infection by multiple species at the sub-microscopic level. The remainder of 153 asymptomatic adults were included in this analysis, of whom 19 had sub-microscopic *P. falciparum* infection, 15 had sub-microscopic *P. vivax* infection, 17 had patent *P. falciparum* infection, and 19 had patent *P. vivax* infection. Fourteen adults with acute uncomplicated malaria diagnosed by microscopy were included for comparison.

Participant characteristics are summarized in Table 1. The majority of groups were gender balanced. Forty-eight per cent of adults (80/153) were Papuan. All participants were resident in the Timika district, with 94 % living in Timika for more than 2 years and 59 % for more than 10 years. Of the 34 asymptomatic adults with sub-microscopic *P. falciparum* or *P. vivax* infection, 91 % (31/34) had lived in Timika for more than 2 years and 56 % (19/34) for more than 10 years. Asymptomatic adults with sub-microscopic *P. falciparum* infection had significantly reduced lymphocyte counts (*P* = 0.015) (Table 1) [14]. There were no other significant differences in baseline characteristics between adults with asymptomatic sub-microscopic infections and controls.

**Table 1:** Study participants

Characteristics	Values for indicated group [median (IQR)] or individual values						
	Control	Asymptomatic				Acute uncomplicated	
		Sub-microscopic	Sub-microscopic	Patent	Patent	Malaria	Malaria
		*P. falciparum*	*P. vivax*	*P. falciparum*	*P. vivax*	*P. falciparum*	*P. vivax*
No. of subjects							
Total	83	19	15	17	19	6	8
Female/male	46/37	10/9	8/7	12/5	14/5	4/2	5/3
Papuan/non-Papuan	29/54	9/10	9/6	7/10	14/5	6/0	6/2
Age (years)	30 (25–37)	31 (26–36)	28 (21–31)	28 (24–36)	32 (30–36)	21 (20–24)*	27 (23–36)
Time resident in area (years)	12 (5–20)	10 (6–18)	9 (5–25)	10 (5–22)	10 (2–15)	NA	NA
No. of parasites/µl	ND	ND	ND	294 (82–2065)	210 (89–696)	24,725 (10,734–57,274)^#^	58,341 (10,778–89,678)^#^
Lymphocyte count (10^9^/l)	2.5 (2.2–3.1)	2.2 (1.5–2.6)^a^	2.6 (2.2–3.7)	2.5 (1.7–2.8)	2.5 (1.8–3.7)	1.4 (0.7–1.6)**	1.0 (0.7–1.2)***
Monocyte count (10^9^/l)	0.5 (0.4–0.6)	0.4 (0.3–0.7)	0.6 (0.4–0.7)	0.6 (0.5–0.7)^a^	0.5 (0.3–0.8)	0.7 (0.6–0.7)*	0.9 (0.4–1.4)^a^

*IQR* interquartile range, *ND* no parasite detected by microscopy, *NA* data not available

Significantly different to control, Kruskal–Wallis test with Dunn’s multiple comparisons

* P < 0.05

** P < 0.005

*** P < 0.0005

^#^Significantly different to species-matched patent infection, Kruskal–Wallis test with Dunn’s multiple comparisons (P < 0.0005)

^a^Significantly different to control, Mann–Whitney *U* test (P < 0.05)

### Peripheral DC HLA-DR expression and DC sub-set numbers are retained in asymptomatic sub-microscopic *Plasmodium falciparum* or *Plasmodium vivax* infection

**Fig. 1:**
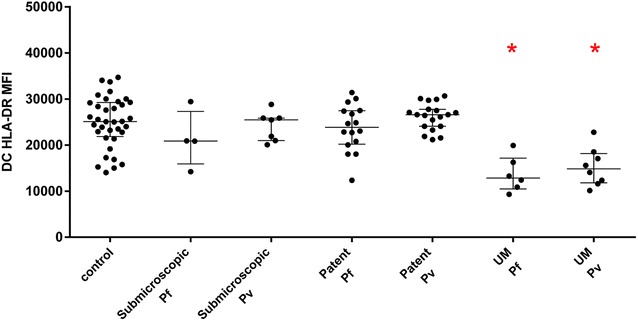
HLA-DR MFI of total DC was determined in adult controls (n = 36), asymptomatic sub-microscopic *P. falciparum* (Pf) infection (n = 4), asymptomatic sub-microscopic *P. vivax* (Pv) infection (n = 7), asymptomatic patent *P. falciparum* infection (n = 16), asymptomatic patent *P. vivax* infection (n = 19), uncomplicated (UM) *P. falciparum*-infected adults (n = 6), and uncomplicated *P. vivax*-infected adults (n = 8). *Graphs* show median with interquartile range. Kruskal–Wallis test with Dunn’s multiple comparisons test was used to compare between groups (*significantly different to control). Data were obtained by analysis of fresh whole blood using 4-colour flow cytometry panels

Total circulating DC was assessed in 54 % (82/153) of participants and identified by flow cytometry as described elsewhere [14] (Additional file 2: Figure S1B). Total DC were defined as lineage marker-negative HLA-DR^+^ cells. The median fluorescent intensity (MFI) of HLA-DR expression was used as a measure of DC activation/maturation. In asymptomatic adults with sub-microscopic and patent *P. falciparum* or *P. vivax* infection, HLA-DR expression on total DC were comparable to controls (Fig. 1). Conversely, as previously reported, adults with acute uncomplicated *P. falciparum* or *P. vivax* malaria had significantly lower expression of HLA-DR on total DC when compared to controls (Fig. 1) [14].

**Fig. 2:**
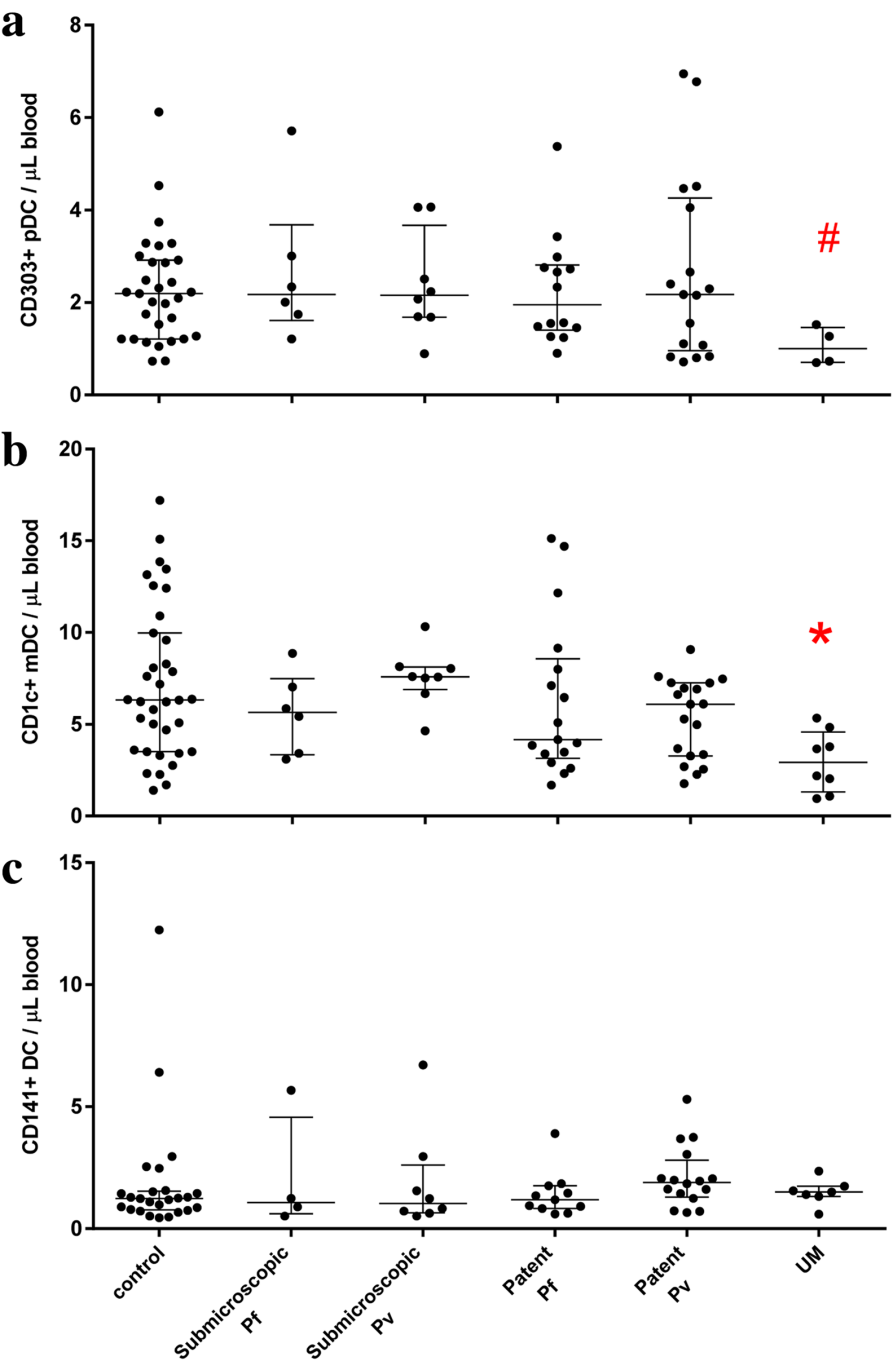
Absolute number of **a** CD303+ plasmacytoid DC (pDC), **b** CD1c+ myeloid DC (mDC) and **c** CD141+ mDC in peripheral blood of controls (**a** n = 31, **b** n = 35 and **c** n = 25), asymptomatic sub-microscopic *P. falciparum* (Pf) infection (**a** n = 6, **b** n = 6 and **c** n = 4), asymptomatic sub-microscopic *P. vivax* (Pv) infection (**a** n = 8, **b** n = 8 and **c** n = 8), asymptomatic patent *P. falciparum* infection (**a** n = 14, **b** n = 17 and **c** n = 11), asymptomatic patent *P. vivax* infection (**a** n = 17, **b** n = 19 and **c** n = 16), and adults with uncomplicated malaria (UM) (**a** n = 4, **b** n = 8 and **c** n = 7). *Graphs* show median with interquartile range. ^#^Kruskal–Wallis test with Dunn’s multiple comparisons test or *Mann–Whitney U test was used to compare between groups (*^#^ significantly different to controls). Data were obtained by analysis of fresh whole blood using 4-colour flow cytometry panels

Circulating DC sub-sets were assessed in 56 % (86/153) of participants and reported as the absolute number of CD303^+^ (blood DC antigen 2 [BDCA-2] positive) plasmacytoid DC (pDC), CD1c^+^ (BDCA-1 positive) mDC (CD1c^+^ mDC), and CD141^+^ (BDCA-3 positive) mDC (CD141^+^ mDC) as per flow cytometric gating of DC sub-sets [14] (Additional file 2: Figure S1A). The number of circulating pDC, CD1c^+^ mDC, and CD141^+^ mDC in asymptomatic adults with either sub-microscopic, or patent *P. falciparum* or *P. vivax* infection were not significantly different from controls (Fig. 2a–c). In contrast, as previously reported [14], pDC and CD1c^+^ mDC numbers were significantly reduced and CD141^+^ mDC were unchanged in adults with acute uncomplicated malaria (Fig. 2a–c). Patients with acute uncomplicated *P. falciparum* or *P. vivax* infection were analysed as a single group because of the low number of patients with DC sub-set data.

### Reduced CD4^+^ T cells and preservation of Treg cells in asymptomatic sub-microscopic *Plasmodium* infection

Regulatory T cells were assessed in 84 % (129/153) of participants and were identified by flow cytometry as CD4^+^CD25^+^CD127^low^ lymphocytes, and sub-divided into CD45RA^−^-activated and CD45RA^+^-resting Treg cells (aTreg and rTreg, respectively) as per flow cytometric gating [14] (Additional file 2: Figure S1C). In asymptomatic adults with sub-microscopic and patent *P. falciparum* or *P. vivax* infection, there were no changes in the absolute or relative number of Treg cells compared to controls (Table 2). In asymptomatic sub-microscopic *P. falciparum* or *P. vivax* infection, the proportion of activated Treg cells (CD25^high^ CD45RA^−^) and rTreg-to-aTreg ratios were comparable to aparasitaemic controls (Table 2). This result differed from the previously reported lower proportion of activated Treg cells in asymptomatic adults with patent *P. falciparum* or *P. vivax* infection [14]. Asymptomatic adults with sub-microscopic *P. falciparum* infection exhibited reduced CD4^+^ T cell numbers in comparison to controls (*P* = 0.009; Table 2).

**Table 2:** T cell data

Parameter	Values for indicated group [median (IQR)] or individual values						
	Control	Asymptomatic				Acute uncomplicated	
		Sub-microscopic	Sub-microscopic	Patent	Patent	Malaria	Malaria
		*P. falciparum*	*P. vivax*	*P. falciparum*	*P. vivax*	*P. falciparum*	*P. vivax*
No. of subjects	70	17	12	12	18	6	8
CD4 T cells/µl blood	752 (611–914)	570 (478–699)^a^	733 (634–1165)	836 (539–1041)	773 (588–941)	201 (117–351)**	243 (170–321)***
CD25^+^ CD127^low^ CD4^+^ Treg cells/µl blood	42 (33–53)	36 (28–46)	44 (40–59)	41 (33–66)	41 (30–54)	14 (11–23)**	16 (11–21)***
% of Treg cells in CD4 T cells	5.4 (4.6–6.2)	6.1 (5.2–7.2)	6.0 (4.1–6.9)	6.3 (5.4–7.1)	5.3 (4.4–6.5)	6.9 (5.7–9.7)	6.3 (5.6–7.4)
CD45RA^−^ CD25^high^ aTreg/µl blood	8.0 (6.1–11.2)	7.7 (5.9–10.2)	8.2 (6.6–12.9)	7.1 (2.8–9.9)	6.0 (5.3–7.3)^a^	3.9 (2.6–5.0)*	4.3 (3.3–7.4)^a^
% of aTreg in total Treg cells	20.7 (16.2–25.8)	23.5 (17.9–28.0)	19.8 (15.3–22.9)	11.9 (8.6–16.4)**	14.8 (13.0–18.1)^a^	25.2 (20.1–33.8)	29.2 (26.3–44.4)*
CD45RA^+^ CD25^+^ rTreg/µl blood	12.3 (6.6–19.3)	8.9 (6.8–13.9)	14.0 (7.6–20.0)	11.7 (9.3–19.3)	10.0 (7.2–17.8)	4.4 (3.1–6.0)*	2.9 (1.7–4.1)***
% of rTreg in total Treg cells	31.7 (20.5–36.9)	29.3 (21.1–35.2)	27.7 (21.1–35.5)	30.8 (22.3–36.2)	28.6 (23.4–38.4)	30.2 (19.1–33.4)	19.4 (14.2–20.5)*
rTreg/aTreg ratio	1.3 (0.9–1.9)	1.0 (0.8–2.0)	1.2 (0.9–2.5)	2.3 (1.4–3.7)*	1.8 (1.3–2.7)^a^	1.1 (0.8–1.5)	0.7 (0.4–0.9)**

*IQR* interquartile range

Significantly different to control, Kruskal–Wallis test with Dunn’s multiple comparisons

* P < 0.05

** P < 0.005

*** P < 0.0005

^a^Significantly different to control, Mann–Whitney *U* test (P < 0.05)

## Discussion

As in previous studies in Southeast Asia [4, 5] a large proportion of adults living in Timika have sub-microscopic infection with *P. falciparum* or *P. vivax*. In these individuals HLA-DR expression on total DC is retained. Similarly, the Treg cell phenotype, characterized by aTreg frequencies and rTreg-to-aTreg ratios, was comparable to that in uninfected adult controls. Collectively, the data indicate that neither DC nor Treg cells are phenotypically dysregulated by asymptomatic sub-microscopic *Plasmodium* infection. The absence of dysregulation suggests functional potential of these cells is maintained and may contribute to the control of infection and clinical immunity to *Plasmodium* infection.

The expression of HLA-DR is required by specialist antigen-presenting cells, such as DC, to present foreign antigen to T cells and is commonly used as a marker for DC activation/maturation. In controlled human *Plasmodium* infection studies, naïve volunteers experimentally infected with *P. falciparum* displayed reduced circulating pDC and mDC levels, followed by down-regulation of HLA-DR on the surface of pDC [22] and CD1c^+^ mDC [23], all occurring at sub-microscopic PCR-level parasitaemia prior to development of patent parasitaemia. Conversely, in both sub-microscopic and patent asymptomatic infections in endemic area residents, the observation of unaltered HLA-DR expression on total circulating DC indicates that DC maturation is not reduced, which may contribute to the maintenance of asymptomatic infection. Comprehensive longitudinal studies in asymptomatic adults are required to fully characterize DC activation/function and to understand their stability and role in the maintenance of sub-microscopic and patent infection.

In contrast to DC activation, Treg cell activation may contribute to progression of disease by suppressing T cell responses and host immunity, favouring parasite growth [20, 21, 28]. CD25^hi^CD45RA^−^-activated Treg cells (aTreg) are highly suppressive and proliferative Treg cells [29], while CD25^+^CD45RA^+^-resting Treg cells (rTreg) provide a reservoir capable of subsequent activation. Adults with asymptomatic sub-microscopic *P. falciparum* or *P. vivax* infection showed neither a reduced nor an over-activated Treg cell response, characterized by aTreg frequencies and rTreg-to-aTreg ratios that were comparable to uninfected adult controls. This contrasted with the reduced Treg cell response characterized by a relative decrease in aTreg frequency and increase in rTreg-to-aTreg ratio in asymptomatic patent *Plasmodium* infection [14]. Furthermore, the data contrast with the more activated Treg cells observed in acute malaria [14, 20]. Therefore, in asymptomatic sub-microscopic infections, the data indicate that the CD45RA phenotype of Treg cells was not modulated and suggests the Treg cell compartment is phenotypically altered only by patent and not sub-microscopic parasitaemia. This suggests maintenance of a normal, stably suppressive environment, allowing other immune cells to contribute to the control of infection.

Correlations between parasitaemia and Treg cells could not be examined since the degree of sub-microscopic parasitaemia was not quantified. Boyle and colleagues [17] recently showed the loss of circulating Treg cells and down-regulation of TNFRII in Ugandan children highly exposed to malaria, which is thought to be implicated in the development of protective immunity to malaria. Additional studies are warranted to examine relationships between Treg cell activation and parasitaemia, including additional Treg cell activation markers (such as TNFRII, CTLA-4), and longitudinal assessment of Treg cell responses in asymptomatic carriers.

In sub-microscopic *P. falciparum* infection, peripheral lymphocytes and CD4^+^ T cells were reduced in asymptomatic adults, a finding similar to that observed in experimental sub-microscopic *P. falciparum* infection of naïve volunteers [22]. Despite this, no change in Treg cell frequency and numbers were found in endemic area residents with asymptomatic sub-microscopic *P. falciparum* or *P. vivax* infection, suggesting that stable Treg cells potentially minimize immune suppression and thereby support immune activation. Although asymptomatic adults with sub-microscopic *P. falciparum* infection were lymphopenic, their lymphocyte and CD4^+^ T cell counts were still significantly higher compared to adults with species-matched acute malaria.

The study had a number of limitations. As patent individuals were treated and sub-microscopic infections were only retrospectively identified, participants could not be longitudinally followed in this study, preventing the assessment of whether infected individuals remained asymptomatic, cleared the infection, or developed symptomatic malaria. It is also acknowledged that the sample sizes in the asymptomatic sub-microscopic groups with DC data were low due to poor sample quality and/or limitations in available blood volume. In addition, PCR was not performed on the sub-set of children in the original study [14] because finger-prick blood volumes were used for whole blood flow cytometry and this left insufficient or no blood for molecular analysis. Despite these limitations, modulations in DC and Treg cells clearly exist between controls, patent infection and acute malaria [14]. The current data indicate that sub-microscopic parasitaemia in endemic adults is not associated with variations in DC HLA-DR or Treg cell CD45RA expression.

## Conclusion

The study provides no phenotypic evidence of DC and Treg cell dysregulation in asymptomatic sub-microscopic *P. falciparum* or *P. vivax* infection. DC in both sub-microscopic and patent infection expressed normal levels of HLA-DR; Treg cells exhibited reduced activation in patent infections and were unaltered in sub-microscopic infections. The lack of apparent phenotypic dysregulation in DC and Treg cells identified in asymptomatic sub-microscopic Indonesian adults contrasts with the compromised DC induced by comparable PCR-level parasitaemia in experimental infection of malaria-naïve volunteers. The data extend the view that these immune cells may contribute to anti-parasitic immune mechanisms. Collectively, the findings suggest that malaria vaccines should aim to ensure appropriate DC and Treg cell responsiveness, a state observed in asymptomatic malaria-endemic area adults with sub-microscopic *Plasmodium* infection, but not found in adult volunteers experiencing first exposure to sub-microscopic *Plasmodium* infection.

## Additional files

10.1186/s12936-016-1382-7 List of antibody panels. The table provided represents the antibody panels used to characterize DC and Treg cells by flow cytometry.
10.1186/s12936-016-1382-7 Representative staining of DC and T cells in fresh whole blood from adults. The illustrations provided represent the flow cytometry gating strategy used to identify DC and Treg cells.

## Abbreviations

DC: dendritic cells
Treg cells: regulatory T cells
HLA-DR: human leukocyte antigen (MHC class II)
PCR: polymerase chain reaction
RBCs: red blood cells
pRBC: parasitized red blood cells
RSMM: Rumah Sakit Mitra Masyarakat
DNA: deoxyribonucleic acid
mDC: myeloid DC
pDC: plasmacytoid DC
