# Supplementary material for: Characterization of blood dendritic and regulatory T cells in asymptomatic adults with sub-microscopic Plasmodium falciparum or Plasmodium vivax infection
Source: Malar J. 2016 Jun 21;15:328. doi: 10.1186/s12936-016-1382-7 (PMC4915178; doi:10.1186/s12936-016-1382-7)
Supplement: Supplementary file 1 — 10.1186/s12936-016-1382-7 List of antibody panels. The table provided represents the antibody panels used to characterize DC and Treg cells by flow cytometry. [file 12936_2016_1382_MOESM1_ESM.docx]

**Additional file 1. List of Antibody Panels**

| **Panel** | **Antibodies (per fluorescent channel)** | | | |
| --- | --- | --- | --- | --- |
|  | **FITC** or **AF488** | **PE** | **PerCP** or **PerCP-Cy5.5** | **APC** or **AF647** |
| DC subsets | CD3, CD14, CD19, CD56 | CD1c | CD303 | CD141 |
| Total DC | CD3, CD14, CD19, CD56 | --- | HLA-DR | --- |
| Treg cells | CD45RA | CD25 | CD4 | CD127 |
