# Supplementary material for: Characterization of blood dendritic and regulatory T cells in asymptomatic adults with sub-microscopic Plasmodium falciparum or Plasmodium vivax infection
Source: Malar J. 2016 Jun 21;15:328. doi: 10.1186/s12936-016-1382-7 (PMC4915178; doi:10.1186/s12936-016-1382-7)
Supplement: Supplementary file 2 — 10.1186/s12936-016-1382-7 Representative staining of DC and T cells in fresh whole blood from adults. The illustrations provided represent the flow cytometry gating strategy used to identify DC and Treg cells. [file 12936_2016_1382_MOESM2_ESM.pptx]

## Slide 1
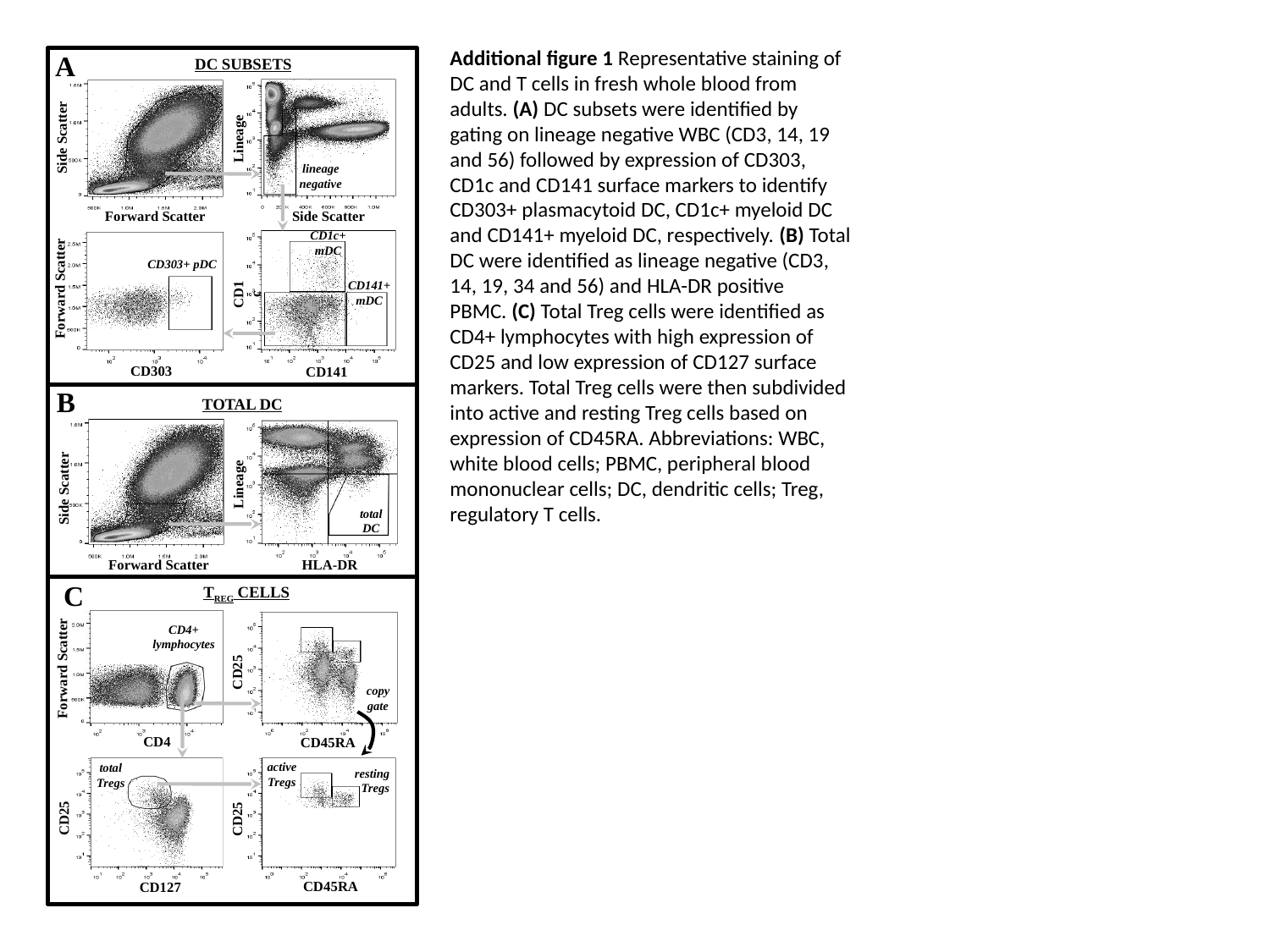

Additional figure 1 Representative staining of DC and T cells in fresh whole blood from adults. (A) DC subsets were identified by gating on lineage negative WBC (CD3, 14, 19 and 56) followed by expression of CD303, CD1c and CD141 surface markers to identify CD303+ plasmacytoid DC, CD1c+ myeloid DC and CD141+ myeloid DC, respectively. (B) Total DC were identified as lineage negative (CD3, 14, 19, 34 and 56) and HLA-DR positive PBMC. (C) Total Treg cells were identified as CD4+ lymphocytes with high expression of CD25 and low expression of CD127 surface markers. Total Treg cells were then subdivided into active and resting Treg cells based on expression of CD45RA. Abbreviations: WBC, white blood cells; PBMC, peripheral blood mononuclear cells; DC, dendritic cells; Treg, regulatory T cells.
A
DC SUBSETS
Lineage
Side Scatter
lineage negative
Forward Scatter
Side Scatter
CD1c+ mDC
CD303+ pDC
CD141+ mDC
Forward Scatter
CD1c
CD303
CD141
B
TOTAL DC
Lineage
Side Scatter
total DC
HLA-DR
Forward Scatter
C
TREG CELLS
CD4+
lymphocytes
Forward Scatter
CD25
copy gate
CD4
CD45RA
active Tregs
total Tregs
resting Tregs
CD25
CD25
CD45RA
CD127
